# Ruxolitinib therapy and telomere length in myelofibrosis

**DOI:** 10.1038/bcj.2016.91

**Published:** 2016-10-07

**Authors:** G Caocci, M Greco, G Delogu, C Secchi, A Perra, S Ghiani, F Orru, A Vacca, F Galimi, G La Nasa

**Affiliations:** 1 Hematology Unit, Department of Medical Sciences, Bone Marrow Transplant Center, R. Binaghi Hospital, University of Cagliari, Cagliari, Italy; 2 Department of Biomedical Sciences, University of Sassari, Sassari, Italy; 3 National Institute of Biostructures and Biosystems, University of Sassari, Sassari, Italy

Telomeres are specialized structures of repetitive nucleotide sequences that cap the ends of human chromosomes. Their main purpose is to maintain genome stability and integrity, and to protect the cell from progressive DNA shortening during repeated division. Human enzyme telomerase complex maintains the length of telomere repeats.^1^ Despite the high levels of telomerase activity in cancer cells, telomere shortening can still occur. Some recent reports describe reduced telomere length in myelofibrosis (MF) regardless of hydroxycarbamide therapy, suggesting a possible prognostic relevance for this biomarker.^2, 3, 4^ As yet, no studies have investigated telomere dynamics following treatment with ruxolitinib, a JAK1/2 inhibitor approved for the treatment of intermediate-2 and high risk MF, primary or post-polycythemia vera (PV) and essential thrombocytemia (ET).^5^

Eight primary MF and three post ET MF patients were given 15 mg or 20 mg of oral ruxolitinib twice daily (BID) depending on baseline platelet counts (100 000/μl to 200 000/μl or >200 000/μl, respectively). The drug dose was escalated to 25 mg BID in patients with an inadequate response and reduced when platelet counts dropped to <100 000/μl. Treatment was stopped when platelet levels fell below 50 000/μl. Telomere lengths were analyzed on unfractionated peripheral blood samples by quantitative PCR (q-PCR) as described by Cawthon^6^ and assessed before and after ruxolitinib at a median of 1000 days (range 113–1152). Primers tel1b(For) 5′-CGG TTT GTT TGG GTT TGG GTT TGG GTT TGG GTT TGG GTT-3′ (270 nM) and tel2b(Rev) 5′-GGC TTG CCT TAC CCT TAC CCT TAC CCT TAC CCT TAC CCT-3′(900 nM) and primers 36B4 36B4u (For) 5′-CAG CAA GTG GGA AGG TGT AAT CC-3′ (300 nM) and 36B4d (Rev) 5′-CCC ATT CTA TCA TCA ACG GGT ACA A-3′ (500 nM) were used for telomere mixture amplification and gene amplification, respectively. The relative telomere length (RTL) was determined as the telomere (T) to single copy gene (36B4) (S) ratio (T/S) normalized to a reference sample (K-562 DNA). Peripheral blood samples were also collected from 11 age-and sex-matched controls from a larger database of 100 healthy subjects.

Median age at diagnosis was 72 years (range 53–83). The JAK2 V617F mutation was detected in seven patients, while CALR and MPL were found in two and one patient, respectively. One patient was triple negative. All patients had splenomegaly with a median enlargement of 17 cm below the costal margin. Based on the IPSS scores, six patients were assigned to the intermediate-2 risk category and five to the high risk category. Ruxolitinib was administered for a median of 1000 days (range 113–1152). Overall, patients received a median of 22 g of ruxolitinib (range 4.6–44.5). All patients showed improvement in constitutional symptoms and quality of life, median weight gain was 7 kg (range 4–14 kg). Splenomegaly decreased by 60% (range 20–100%). Related samples Wilcoxon signed-rank test performed before treatment with ruxolitinib showed that the mean RTL was shorter in patients compared with age-and sex-matched healthy controls (1.08 vs 1.26, respectively; *P*=0.09). After treatment, median RTL increased significantly (1.30 vs 1.08; *P*=0.018), showing overlapping values with the healthy controls (Figure 1). Median RTL elongation from baseline was 15%. Univariate and multivariate analyses included the following parameters: primary MF, presence of the JAK2 V617F mutation, high IPSS score, a decrease in splenomegaly of >50%, >50% bone marrow (BM) cellularity before and after treatment, duration of treatment >1000 days and total drug dose of >22 g. Variables with a *P*-value lower than 0.2 in univariate analysis were included in multivariate analysis using a multi-step forward binary logistic regression model, where RLT >15% from baseline was considered a dependent variable. Only pretreatment BM cellularity of >50% significantly correlated with >15% telomere elongation (*P*=0.004).

In our small cohort of patients, telomere length was restored to normal values after treatment with ruxolitinib. Our observation could stem from a non-specific anti-cytokine action or qualitative changes in clonal hematopoiesis. Indeed, it is possible that ruxolitinib mediates modulation of the BM microenvironment, thereby stimulating stem cell hematopoiesis.^7^ Moreover, It has been demonstrated that oxidative stress and inflammation contributes to a significant decrease in telomerase activity causing telomere shortening.^8^ Ruxolitinib suppresses proinflammatory cytokines through interference with JAK-signal transducer and activator of transcription (STAT) signaling, and thus reverses a potential mechanism of telomere shortening. Despite the uniqueness of this study some limitations need to be noted. First of all, the cohort of patients was relatively small and we did not determine sorted myeloid compartment telomere length. Our preliminary observations need to be validated in the context of controlled studies to achieve new insights on the role of treatment with telomerase inhibitors in MF patients.^9^

## Figures and Tables

**Figure 1:**
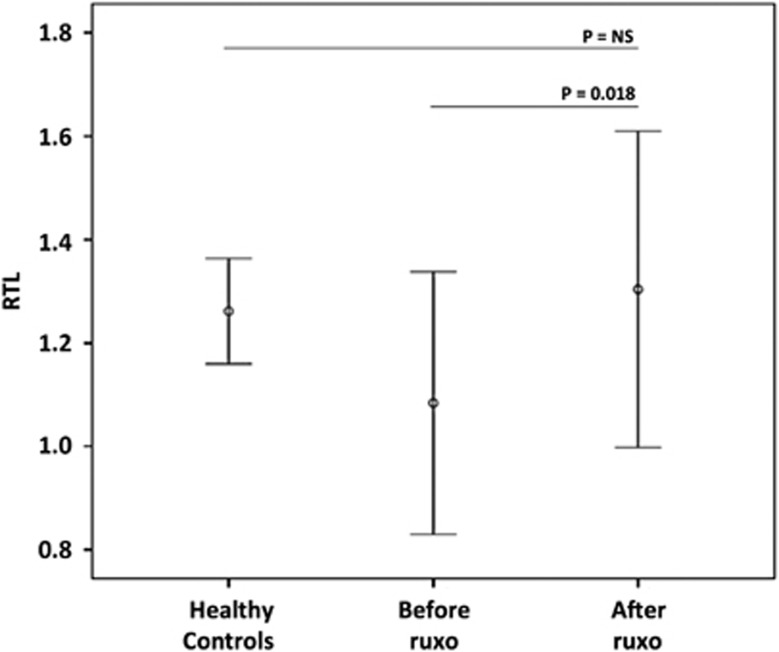
Relative telomere lengths (RTL) before and after ruxolitinib treatment in 11 patients and a group of age- and sex-matched healthy controls. Ruxo=ruxolitinib; NS=not significant.
